# Comparative risk of dementia between direct oral anticoagulants and warfarin after atrial fibrillation related ischemic stroke

**DOI:** 10.3389/fnagi.2026.1718536

**Published:** 2026-04-23

**Authors:** Sangwon Choi, Sojeong Park, Young Hee Jung, Mi Sun Oh, Kyung-Ho Yu, Byung-Chul Lee, Minwoo Lee

**Affiliations:** 1 Department of Neurology, Hallym University Sacred Heart Hospital, Anyang, South Korea; 2 Department of Data Science, Hanmi Pharm. Co., Ltd, Seoul, South Korea

**Keywords:** atrial fibrillation, dementia, direct oral anticoagulant, ischemic stroke, Warfarin

## Abstract

**Introduction:**

Direct oral anticoagulants (DOAC) have been associated with a reduced risk of dementia compared to warfarin in patients with atrial fibrillation (AF) without prior stroke. However, the impact of DOAC on dementia risk in AF-related ischemic stroke survivors is unclear.

**Methods:**

We conducted a retrospective, nationwide cohort study using the Korean National Health Insurance Service database. We identified patients with newly diagnosed ischemic stroke and concurrent AF who began DOAC or warfarin therapy within one month after stroke. Incidence of all-cause dementia, Alzheimer's dementia (AD), and vascular dementia (VaD) was compared between groups using multivariable Cox models with inverse probability of treatment weighting.

**Results:**

A total of 3,112 patients (mean age 70.6 ± 9.5 years; 66.6% male) were analyzed, including 2,919 DOAC users and 193 warfarin users. Over a mean follow-up of 3.63 years, 673 all-cause dementia cases (538 AD, 168 VaD) occurred. After IPTW, DOAC use was associated with higher risks of all-cause dementia (HR 1.16, 95% CI 1.04–1.30) and AD (HR 1.85, 95% CI 1.62–2.13) but a lower risk of VaD (HR 0.54, 95% CI 0.45–0.66) compared to warfarin.

**Discussion:**

In this retrospective nationwide cohort of AF-related ischemic stroke survivors, DOAC use was associated with a higher incidence of all-cause dementia and Alzheimer's dementia, but a lower incidence of vascular dementia, compared with warfarin. These observational findings suggest that anticoagulant type may be differentially associated with subsequent dementia subtypes in this high-risk population and should be interpreted with caution.

## 1 Introduction

Atrial fibrillation (AF) is a common and growing concern, particularly among aging populations (Oltman et al., 2024; Linz et al., 2024), significantly elevating the risk of both ischemic stroke and cognitive decline (Wolf et al., 1991; Kalantarian et al., 2013). AF is thought to increase dementia risk directly through subclinical cerebrovascular injury such as silent infarctions and microbleeds (Kühne et al., 2022; Rivard et al., 2022). Because ischemic stroke survivors already face substantially elevated risks of cognitive impairment and dementia (Corraini et al., 2017; Mijajlovic et al., 2017), the coexistence of AF and prior stroke places patients at even higher risk. Accordingly, optimal management in AF-related ischemic stroke patients is particularly important for long-term cognitive health.

Oral anticoagulants (OAC), notably warfarin and direct oral anticoagulants (DOAC), are standard therapies for preventing thromboembolic events in AF patients with higher risk of cardioembolism (Stroke Prevention in Atrial Fibrillation Investigators, 1991; Giugliano et al., 2013; Patel et al., 2011; Granger et al., 2011; Connolly et al., 2009). Recent guidelines now recommend DOAC over warfarin in preventing stroke except for those with concurrent valvular diseases (Joglar et al., 2024). Apart from the stroke recurrence risk reduction, additional evidence has suggested that DOAC may offer further benefits by reducing the long-term risk of cognitive decline and dementia in patients with AF (Fong et al., 2023). Although some studies have included patients with prior stroke, much of the existing evidence regarding DOAC and dementia risk has been derived from AF cohorts without a primary focus on stroke survivors. Whether these associations hold in AF-related ischemic stroke patients—who differ meaningfully in their vascular injury burden—remains unclear. Moreover, dementia subtypes such as Alzheimer's dementia (AD) and vascular dementia (VaD) have been seldom evaluated separately.

Therefore, we examined the comparative associations of DOAC and warfarin with risks of all-cause dementia, AD, and VaD in AF-related ischemic stroke survivors. Importantly, this higher-risk group allows us to evaluate whether the patterns reported in general AF cohorts manifest similarly—or differently—among patients with substantially greater vulnerability.

## 2 Methods

### 2.1 Data source and study population

This study was designed as a retrospective, nationwide, population-based cohort study using data from 2016 to 2019 in the Korean National Health Insurance Service (K-NHIS) database, which contains routinely collected health claims and health screening information for nearly the entire Korean population (Cheol Seong et al., 2017; Kyoung and Kim, 2022). South Korea operates a national single-payer healthcare system that covers more than 97% of the entire population. The K-NHIS database consists of medical claims records, detailed information on drug prescriptions, healthcare facility usage, demographic details, diagnostic codes according to the ICD-10 codes. Further, the K-NHIS recommends biennial health check-ups free of charge for all adults aged 40 or older, providing laboratory values, lifestyle questionnaire results, and physical assessments (Kim et al., 2022).

**Figure 1:**
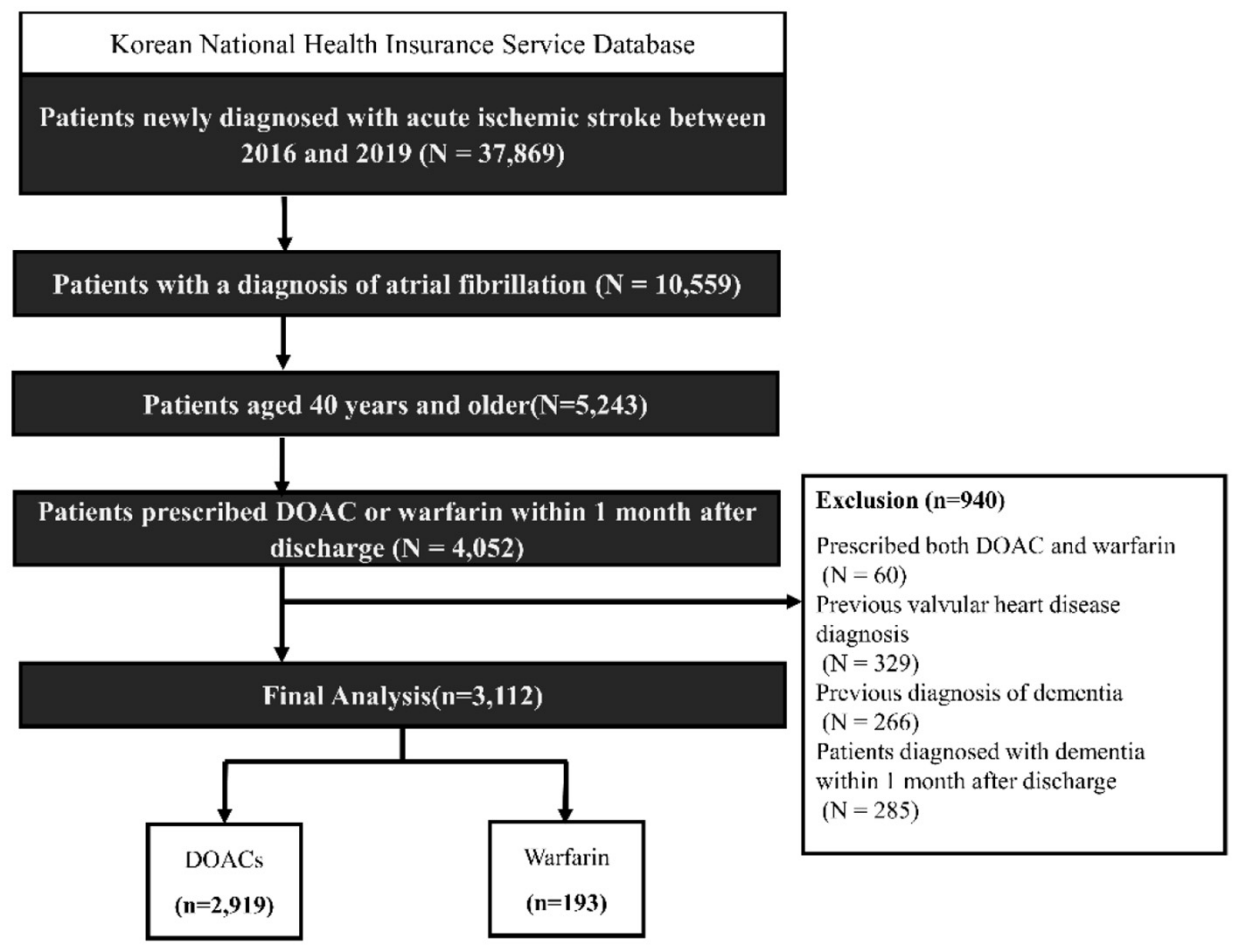
Study flowchart.

We identified a total of 37,869 patients who were newly diagnosed with acute ischemic stroke between 2016 and 2019. Of these, 27,310 patients without a diagnosis of atrial fibrillation were excluded. We then excluded patients who were under 40 years of age (*n* = 5,316) and did not receive a prescription for either DOAC or warfarin within1 month after discharge (*n* = 1,191). An additional 60 patients who were prescribed both DOAC and warfarin were excluded. We also excluded patients with valvular heart disease (*n* = 329), prior diagnosis of dementia (*n* = 266), and diagnosed with dementia within1 month after discharge (*n* = 285). After applying all exclusion criteria, the final analytic cohort consisted of 3,112 patients (Figure 1). Patients with ischemic stroke were identified based on ICD-10 codes (I63–I64) combined with hospitalization records involving brain imaging (CT or MRI), following methods validated in previous studies utilizing the K-NHIS database (Kang et al., 2017).

### 2.2 Main exposure and study outcomes

The main exposure was the type of oral anticoagulant prescribed, categorized as DOAC or warfarin. The DOAC group included patients prescribed dabigatran, apixaban, rivaroxaban, or edoxaban. In the K-NHIS database, drug-level identification depends on the availability of generic formulations. During the study period, only rivaroxaban was individually identifiable, whereas apixaban, edoxaban, and dabigatran were provided as a grouped category. Accordingly, individual DOAC agents could not be distinguished, and DOACs were analyzed as a single class.

The primary study outcome was the incident of all-cause dementia, which encompassed both AD and VaD along with other types of dementia. All-cause dementia was defined by ICD-10 codes (F00–F03, G30, G31) accompanied by prescription records for anti-dementia medications (donepezil, galantamine, rivastigmine, or memantine). Secondary outcomes, including Alzheimer's dementia (AD; F00, G30) and vascular dementia (VaD; F01), were similarly defined.

### 2.3 Definition of variables

We used each participant's initial health screening data as baseline information. Baseline age was defined as the age at the time of enrollment. Body mass index (BMI) was calculated by dividing weight in kilograms by the square of height in meters. Smoking status was classified as never smoker, former smoker, or current smoker. Alcohol consumption was categorized into three groups: none, mild (ethanol intake < 30 g/day for men and < 20 g/day for women), and heavy (ethanol intake ≥30 g/day for men and ≥20 g/day for women). Regular exercise was defined as engaging in vigorous exercise at least three times per week or moderate exercise at least five times per week (Lee et al., 2020). Income was categorized into quartiles, and individuals in the lowest quartile were included as a covariate. Medical comorbidities were identified based on ICD-10 diagnostic codes. Hypertension was defined by codes I10–I15, diabetes mellitus by codes E10–E14, and dyslipidemia by code E78. Chronic kidney disease (CKD) was identified using codes N18, and depression was captured using codes F32–F33.

### 2.4 Statistical analysis

Baseline characteristics between the DOAC and warfarin groups were summarized using means and standard deviations for continuous variables and percentages for categorical variables. Differences between groups before weighting were evaluated using absolute standardized differences (ASDs). Given the differences in baseline characteristics and sample sizes between the DOAC and warfarin groups, inverse probability of treatment weighting (IPTW) based on propensity scores was applied to reduce potential selection bias and confounding (Austin, 2009). Propensity scores were estimated using logistic regression models with anticoagulant type (DOAC versus warfarin) as the dependent variable. Covariates were selected a priori based on established associations with anticoagulant prescribing and dementia risk. The covariates included age, sex, income level, BMI, smoking status, alcohol consumption, regular exercise, hypertension, diabetes mellitus, dyslipidemia, CKD, depression, and CHA_2_DS_2_-VASc score. Multicollinearity among covariates was evaluated using variance inflation factors, and no concerning multicollinearity was observed. Covariate balance before and after weighting was assessed using absolute standardized differences (ASDs), with an ASD < 0.1 considered adequate. Stabilized weights were used, and no extreme weights requiring truncation were observed. A covariate balance plot (Supplementary Figure S1) is provided to visually summarize balance improvement after IPTW.

Crude incidence rates (IRs) of dementia outcomes were calculated per 1,000 person-years. Cox proportional hazards models were used to estimate hazard ratios (HRs) and 95% confidence intervals (CIs) for the association between anticoagulant type and dementia outcomes, both in unadjusted models and in models adjusted for age, sex, and other clinical covariates. Furthermore, weighted Cox proportional hazard models using IPTW-adjusted cohorts were additionally applied to provide robust estimates controlling for residual confounding. Cumulative incidence curves for all-cause dementia, AD, and VaD were generated using the Kaplan–Meier method, and differences between groups were assessed using the log-rank test. Subgroup analyses were performed to evaluate potential effect modification by baseline characteristics and comorbidities. All statistical analyses were conducted using SAS version 9.4 (SAS Institute Inc., Cary, NC). Two-sided *P*-values less than 0.05 were considered statistically significant.

## 3 Results

### 3.1 Baseline demographic findings

A total of 3,112 patients were included in the study, with a mean follow-up duration of 3.63 ± 1.95 years. Of these, 2,919 patients were treated with DOAC and 193 with warfarin. The mean age was similar between the two groups (70.66 ± 9.53 years vs. 70.22 ± 9.70 years). Significant differences were observed between the two groups. Current smoking was more prevalent in the warfarin group, whereas alcohol consumption and regular exercise were more common in the DOAC group. The CHA_2_DS_2_-VASc score and prevalence of chronic kidney disease were higher in the warfarin group. Overall, these differences—particularly in CKD and CHA_2_DS_2_-VASc—indicate meaningful baseline imbalance prior to weighting. Given the difference in sample sizes and baseline characteristics between the two groups, inverse probability of treatment weighting (IPTW) was applied to minimize group differences. After IPTW, differences were adequately minimized, with all ASD reduced to below 0.1 (Table 1, Supplementary Figure S1). But, the relatively small number of warfarin users may contribute to wider confidence intervals and lower precision of the estimated associations, despite improved covariate balance after IPTW.

**Table 1:** Baseline characteristics of the patients included in the study.

Variables	Before IPTW			After IPTW		
	DOAC (*n* = 2,919)	Warfarin (* n* = 193)	ASD	DOAC	Warfarin	ASD
Age (years)	70.66 ± 9.53	70.22 ± 9.70	0.05	70.63 ± 9.87	70.48 ± 38.51	0.01
Male	1,948 (66.74%)	123 (63.73%)	0.06	66.54%	67.72%	0.02
BMI (kg/m^2^)	24.66 ± 3.31	24.39 ± 3.25	0.08	24.64 ± 3.41	24.79 ± 14.41	0.05
BMI 5 level			0.09			0.09
BMI < 18.5	68 (2.33%)	4 (2.07%)		2.32%	2.70%	
BMI 18.5– < 23	804 (27.54%)	58 (30.05%)		27.71%	26.45%	
BMI 23– < 25	762 (26.10%)	53 (27.46%)		26.18%	26.80%	
BMI 25– < 30	1,111 (38.06%)	69 (35.75%)		37.90%	36.26%	
BMI ≥ 30	174 (5.96%)	9 (4.66%)		5.89%	7.79%	
Income, low	622 (21.31%)	46 (23.83%)	0.06	21.48%	20.01%	0.04
Smoking			0.21			0.04
Non	1730 (59.27%)	118 (61.14%)		59.39%	57.60%	
Ex	880 (30.15%)	44 (22.80%)		29.68%	31.41%	
Current	309 (10.59%)	31 (16.06%)		10.93%	10.99%	
Drinking			0.18			0.06
Non	1,899 (65.06%)	141 (73.06%)		65.53%	62.48%	
Mild	770 (26.38%)	40 (20.73%)		26.05%	28.47%	
Heavy	250 (8.56%)	12 (6.22%)		8.42%	9.06%	
Regular exercise	802 (27.48%)	42 (21.76%)	0.13	27.15%	26.91%	0.01
Depression	308 (10.55%)	23 (11.92%)	0.04	10.64%	9.68%	0.03
Diabetes mellitus	1,184 (40.56%)	86 (44.56%)	0.08	40.83%	39.10%	0.04
Hypertension	2,483 (85.06%)	166 (86.01%)	0.03	85.13%	84.63%	0.01
Dyslipidemia	2,039 (69.85%)	141 (73.06%)	0.07	70.08%	72.07%	0.04
Chronic kidney disease	125 (4.28%)	37 (19.17%)	0.48	5.18%	4.64%	0.02
CHA_2_DS_2_-VASc score	3.06 ± 1.78	3.30 ± 1.81	0.13	3.07 ± 1.85	3.03 ± 7.16	0.03
SBP (mmHg)	129.00 ± 16.57	129.40 ± 17.02	0.02	129.03 ± 17.15	129.11 ± 65.52	0.01
DBP (mmHg)	78.37 ± 11.32	77.52 ± 10.69	0.08	78.32 ± 11.67	78.69 ± 40.89	0.03
HDL-C (mg/dL)	51.89 ± 13.20	51.60 ± 12.78	0.02	51.88 ± 13.63	52.72 ± 50.81	0.06
LDL-C (mg/dL)	93.97 ± 35.55	92.86 ± 35.01	0.03	93.91 ± 36.73	94.49 ± 138.90	0.02
Fasting glucose (mg/dL)	108.90 ± 29.16	108.10 ± 30.14	0.03	108.86 ± 30.04	106.24 ± 102.38	0.09
Total cholesterol (mg/dL)	170.30 ± 40.21	169.10 ± 39.71	0.03	170.28 ± 41.53	171.30 ± 154.96	0.03
Triglyceride^*^ (mg/dL)	108.15 (106.22–110.11)	109.03 (101.54–117.07)	0	108.20 (106.27–110.16)	106.54 (98.48–115.26)	0.04

^*^Geometric mean (95% CI).

Abbreviation: BMI, body mass index; BP, blood pressure; HDL-C, high density lipoprotein cholesterol; LDL-C, low density lipoprotein cholesterol; GFR, glomerular filtration rate.

### 3.2 Association of anticoagulation and dementia

The crude IR of all-cause dementia was 48.63 per 1,000 person-years in the warfarin group and 60.26 in the DOAC group. For AD, crude IR was 27.76 in warfarin group and 46.76 in DOAC group. In the Cox proportional hazards model adjusted for age, sex, and other clinical covariates, DOAC use was not significantly associated with the risk of all-cause dementia (adjusted HR 1.17, 95% CI 0.83–1.65) or VaD (adjusted HR 0.60, 95% CI 0.35–1.04). In contrast, DOAC use was associated with a higher risk of AD, with an adjusted hazard ratio of 1.66 (95% CI 1.08–2.56); the crude incidence rates were 46.76 and 27.76 per 1,000 person-years in the DOAC and warfarin groups, respectively. After applying IPTW, DOAC use compared to warfarin was associated with a significantly higher risk of all-cause dementia (HR 1.16, 95% CI 1.04–1.30; *p* = 0.009) and AD (HR 1.85, 95% CI 1.62–2.13; *p* < 0.001), but a significantly lower risk of VaD (HR 0.54, 95% CI 0.45–0.66; *p* < 0.001) (Table 2). Although the relative increase in all-cause dementia associated with DOAC compared with warfarin was modest, the association with Alzheimer's dementia was substantially larger, whereas DOAC use was associated with a lower risk of vascular dementia.

**Table 2:** Outcomes before IPTW and after IPTW according to the anticoagulation.

Outcome	OAC	*N*	Events	IR (per 1,000 PY)	Crude HR (95% CI)	Adjusted HR (95% CI)	After IPTW HR (95% CI)	*P* value
Primary outcome								
All-cause dementia	Warfarin	193	36	48.63	1 (ref)	1 (ref)	1 (ref)	0.009
	DOAC	2,919	637	60.26	1.19 (0.85–1.66)	1.17 (0.83–1.65)	1.16 (1.04–1.30)	
Secondary outcomes								
Alzheimer's dementia	Warfarin	193	22	27.76	1 (ref)	1 (ref)	1 (ref)	< 0.0001
	DOAC	2,919	516	46.76	1.63 (1.06–2.49)	1.66 (1.08–2.56)	1.85 (1.62–2.13)	
Vascular dementia	Warfarin	193	15	19.11	1 (ref)	1 (ref)	1 (ref)	< 0.0001
	DOAC	2,919	153	13.07	0.64 (0.38–1.09)	0.60 (0.35–1.04)	0.54 (0.45–0.66)	

Abbreviations: OAC, oral anticoagulation; DOAC, direct oral anticoagulation; IR, incidence rate; IPTW, inverse probability of treatment weighting.

Adjusted for age, sex, income, BMI, smoking, drinking, regular exercise, hypertension, dyslipidemia, diabetes mellitus, chronic kidney disease, and depression.

**Figure 2:**
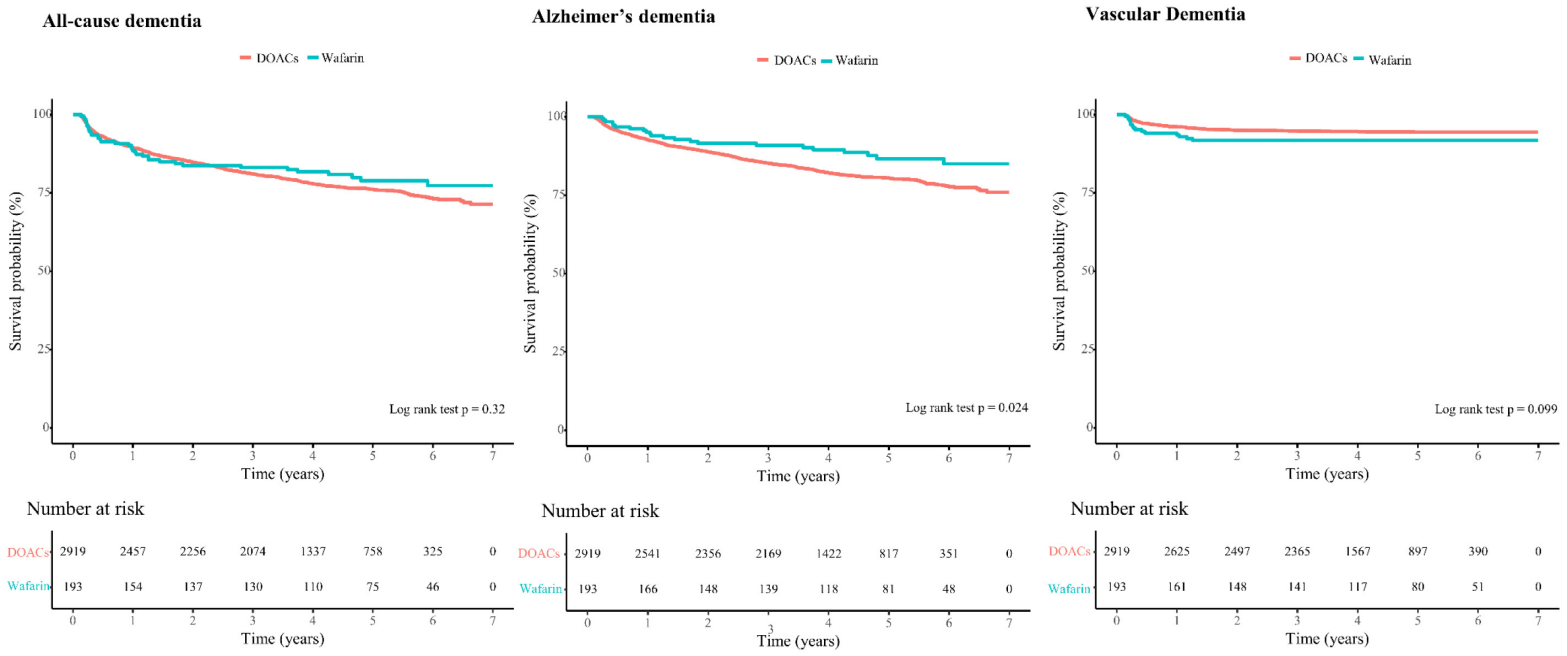
Cumulative incidence curves of each outcome were compared according to anticoagulants using a log-rank test.

Cumulative incidence curves for all-cause dementia, AD, and VaD stratified by anticoagulant type are illustrated in Figure 2.

### 3.3 Subgroup analysis

Prespecified subgroup analyses of the association between OAC type and incident dementia risk are presented in Table 3. No statistically significant interactions were observed for all-cause dementia and AD across the subgroups. For VaD, a significant risk reduction associated with DOAC use was observed in patients with low income (HR 0.188, 95% CI 0.079–0.451, *p* < 0.05). HR in non-hypertensive patients could not be reliably determined due to small sample sizes and event counts.

**Table 3:** Subgroup analyses of outcomes according to demographics factor and comorbidities.

Subgroup	Category	All-cause dementia aHR (95% CI)	*P* for interaction	AD aHR (95% CI)	*P* for interaction	VaD aHR (95% CI)	*P* for interaction
Sex	Male	1.09 (0.69, 1.72)	0.676	1.35 (0.77, 2.38)	0.3886	0.66 (0.31, 1.37)	0.768
	Female	1.28 (0.76, 2.15)		2.13 (1.08, 4.18)		0.54 (0.24, 1.22)	
Age	≥60	1.18 (0.83, 1.69)	0.7874	1.68 (1.08, 2.62)	0.7128	1.27 (0.15, 10.50)	0.6676
	< 60	1.20 (0.27, 5.30)		1.38 (0.16, 11.54)		0.57 (0.33, 1.00)	
Income, low	No	1.18 (0.80, 1.75)	0.5907	1.36 (0.87, 2.14)	0.1454	0.91 (0.44, 1.89)	0.0382
	Yes	1.04 (0.50, 2.15)		5.61 (1.32, 23.80)		0.19 (0.08, 0.45)	
Obesity (BMI ≥25)	No	1.17 (0.75, 1.83)	0.7423	1.44 (0.86, 2.44)	0.4664	0.72 (0.34, 1.50)	0.5472
	Yes	1.27 (0.73, 2.19)		2.36 (1.09, 5.12)		0.53 (0.23, 1.20)	
Diabetes mellitus	No	0.98 (0.62, 1.53)	0.368	1.73 (0.94, 3.18)	0.8985	0.40 (0.21, 0.74)	0.0529
	Yes	1.58 (0.92, 2.71)		1.74 (0.93, 3.24)		1.54 (0.47, 5.04)	
Hypertension	No	1.69 (0.40, 7.15)	0.404	1.37 (0.31, 5.94)	0.9522	#	
	Yes	1.15 (0.81, 1.65)		1.73 (1.10, 2.72)		0.54 (0.31, 0.93)	
Dyslipidemia	No	1.14 (0.54, 2.41)	0.6786	1.53 (0.60, 3.93)	0.9516	0.74 (0.22, 2.49)	0.4458
	Yes	1.17 (0.80, 1.73)		1.72 (1.05, 2.81)		0.55 (0.30, 1.01)	
Depression	No	1.07 (0.74, 1.54)	0.2341	1.53 (0.96, 2.43)	0.431	0.57 (0.32, 1.00)	0.4156
	Yes	2.28 (0.81, 6.41)		2.93 (0.90, 9.55)		1.47 (0.18, 11.84)	

#Hazard ratio for VaD in non-hypertensive patients could not be reliably determined due to small sample sizes and event counts.

## 4 Discussion

In this large, nationwide retrospective cohort of AF-related ischemic stroke, we observed a differential association between oral anticoagulant type and dementia subtypes. Compared with warfarin, DOAC use was associated with a significantly higher risk of all-cause dementia and AD, but a significantly lower risk of vascular dementia (HR 0.54). This finding indicates that patients treated with warfarin had a relatively higher incidence of VaD in this cohort. Subgroup analyses showed generally consistent associations between anticoagulant type and dementia outcomes, with no statistically significant interactions observed except for VaD with low income, where DOAC use was associated with a significantly lower risk.

Our findings contrast notably with prior research conducted in the general AF population without stroke. For example, previous study demonstrated a lower dementia risk associated with DOAC use compared to warfarin in AF patients without a prior stroke history (Lee et al., 2021). However, our study, specifically targeting AF-related ischemic stroke survivors, revealed a different pattern: DOAC use was associated with a significantly higher risk of all-cause dementia, AD and showed a lower risk of VaD. It suggests that potential differences in dementia risk profiles between the general AF population and those with prior stroke.

Several hypotheses may explain these differential outcomes. First, post-stroke patients inherently carry a higher burden of vascular injury, inflammation, and compromised cerebral integrity, potentially altering how anticoagulants impact cognitive decline (Rost et al., 2022; El Husseini et al., 2023). Prior literature has described potential links between warfarin, vitamin K—dependent coagulation factors such as thrombin, and AD-related biological pathways. Thrombin has been discussed as a possible contributor to neuroinflammation and amyloid-related processes in AD (Grammas and Martinez, 2014; Iannucci et al., 2020; Iannucci and Grammas, 2023). Prior studies examining thrombin inhibition, including those involving dabigatran, have noted reductions in amyloid deposition, and inflammation-related processes, although the clinical relevance of these findings remains uncertain (Grossmann, 2021; Bihaqi et al., 2021). Similar thrombin-related pathways have been considered in the context of warfarin, but these remain conceptual and cannot be examined using our data.

Additionally, warfarin-treated patients might represent a distinct subgroup characterized by closer healthcare monitoring due to the requirement of frequent INR checks. This regular clinical follow-up could facilitate early identification and better management of cognitive impairment and other comorbidities, potentially reducing dementia risk indirectly. Indeed, good INR control among VKA users has been associated with a 27% reduction in dementia risk compared to poor INR control (Cadogan et al., 2021). Conversely, DOAC users, benefiting from fewer healthcare interactions, might experience delayed detection and intervention for subtle cognitive changes, possibly contributing to the higher observed dementia incidence in our analysis. However, these interpretations are speculative and should be considered hypothesis-generating, as our claims-based dataset does not include biological or imaging measures needed to directly evaluate these pathways.

The observed reduction in VaD risk with DOAC use aligns well with the known benefits of DOAC in providing stable and consistent anticoagulation, thereby reducing recurrent cerebrovascular events, microinfarcts, and cerebral microbleeds compared to warfarin (Umemura et al., 2020; Carnicelli et al., 2022; Chen et al., 2018). Prior studies have linked warfarin's variability in anticoagulation, reflected by frequent INR fluctuations, with increased vascular dementia risk (Bunch et al., 2016; Jacobs et al., 2014).

This study has several limitations that should be considered when interpreting the findings. First, dementia diagnoses relied on ICD-10 diagnostic codes coupled with dementia medication prescriptions. Although this method is validated and commonly used in large-scale studies (Lee et al., 2021; Bhattacharyya et al., 2025; Hwangbo et al., 2023), it may still result in misclassification or underdiagnosis, as detailed neuropsychological assessments were unavailable. Second, key clinical measures such as index stroke severity including National Institutes of Health Stroke Scale (NIHSS) score, infarct volume, lesion location were not available in the claims data. We therefore could not adjust for stroke severity, which is a major determinant of post-stroke cognitive trajectories and may also influence anticoagulant selection. In addition, warfarin anticoagulation quality—particularly time in therapeutic range—could not be assessed in this administrative dataset. Because both stroke severity and INR control strongly affect dementia risk and treatment effectiveness, the absence of these variables may introduce residual confounding. Third, as described in the Methods, detailed information on individual DOAC agents was limited by the structure of the NHIS database. Accordingly, we were unable to perform comparative analyses across DOACs. Such analyses would be clinically informative, as potential differences in pharmacologic profiles may influence dementia risk, and should be explored in future studies. Fourth, despite employing IPTW to minimize confounding, residual confounding or selection bias remains possible—particularly given the relatively small number of warfarin users and the absence of key clinical variables such as cognitive reserve, baseline cognitive status, and anticoagulation quality. In addition, IPTW creates a weighted pseudo-population in which covariate distributions are balanced between treatment groups. When treatment groups differ in size, as in the present study, a limited number of individuals in the smaller group may receive relatively larger weights, potentially amplifying their influence in the weighted analysis. Although stabilized weights were applied and no extreme weights were observed, weighting may still influence the apparent associations of certain covariates. Therefore, the IPTW-adjusted estimates should be interpreted cautiously. Fifth, differences in healthcare utilization between warfarin and DOAC users may also introduce differential surveillance bias, potentially influencing the likelihood of dementia detection rather than true incidence. Moreover, the mean follow-up duration of 3.6 years is relatively short for capturing the development of AD with long prodromal periods, and our findings may therefore underestimate AD risk. Sixth, given the observational nature of this study, the findings should not be interpreted as causal. Finally, this study was conducted exclusively in a Korean population within a single-payer healthcare system. Differences in genetic background, healthcare access, and treatment practices across regions may influence both anticoagulant prescribing patterns and dementia risk. Therefore, caution is needed in applying these findings to other populations.

## 5 Conclusion

In conclusion, among AF-related ischemic stroke patients, DOAC use was associated with increased risks of all-cause dementia and Alzheimer's disease, but lower risk of vascular dementia compared to warfarin. These findings suggest that anticoagulant type may be differentially associated with dementia outcomes in this specific population. Further research is needed to confirm our results and to better inform clinical decisions regarding anticoagulant selection.

## Data Availability

The data analyzed in this study is subject to the following licenses/restrictions: The data from the Korean National Health Insurance Service (NHIS) can be accessed via the Health Insurance Data Service website (http://nhiss.nhis.or.kr). Researchers should submit a study proposal for approval from each institutional review board, which is reviewed by the NHIS review committee, to access the database. Requests to access these datasets should be directed to http://nhiss.nhis.or.kr.
