# Supplementary figures and images for: Comparative risk of dementia between direct oral anticoagulants and warfarin after atrial fibrillation related ischemic stroke
Source: Front Aging Neurosci. 2026 Apr 23;18:1718536. doi: 10.3389/fnagi.2026.1718536 (PMC13149358; doi:10.3389/fnagi.2026.1718536)

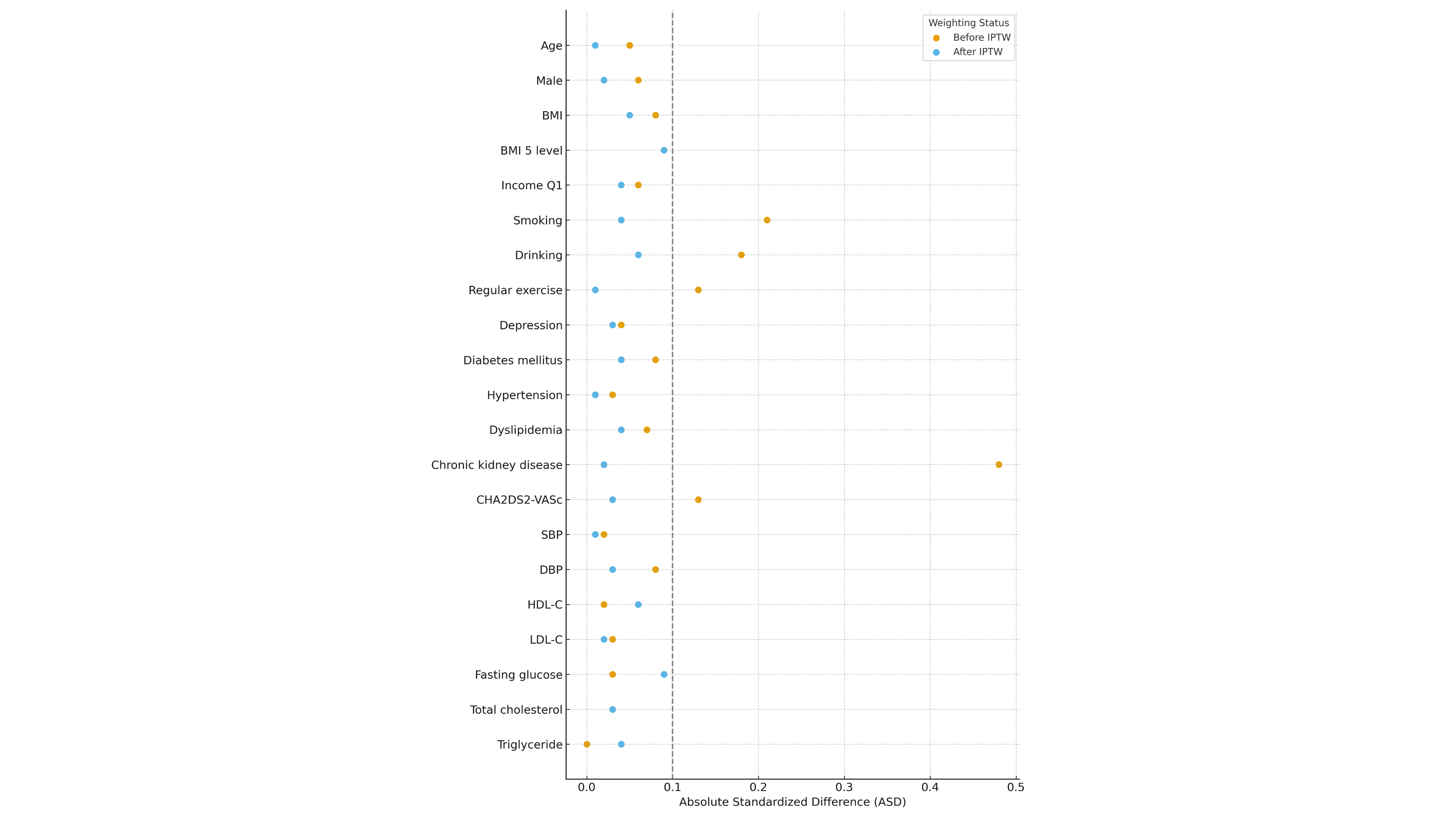

Supplement: Supplementary file 1 [file Image_1.tif]
